# Editorial: Role of the surface in peri-implant bone biology

**DOI:** 10.3389/fbioe.2023.1340681

**Published:** 2023-11-30

**Authors:** Antonio López-Valverde, Tiago Borges, Francisco Vale, Bruno Macedo De Sousa

**Affiliations:** ^1^ Department of Surgery, University of Salamanca, Salamanca, Spain; ^2^ Department of Surgery, Universidad Católica Portuguesa, Viseu, Portugal; ^3^ Department of Orthodontics, University of Coimbra, Coimbra, Portugal; ^4^ Department of Occlusion, University of Coimbra, Coimbra, Portugal

**Keywords:** dental implant, surface, peri-implant and periodontal bone, biology, Osseointegragion

With consistent results and a high success rate in the rehabilitation of edentulous spaces, dental implant surgery has become a very reliable therapeutic option in the 21st century. The rapid osseointegration of the devices allows prosthetic rehabilitations in a shorter period of time and the approach to complex cases with greater safety in the results. In spite of all these favorable expectations, a certain number of implants fail in a short time, either due to a bad praxis on the part of the professional, or due to poor care or maintenance by the patient, or due to factors related to the implant surface that conditions the bone morphology around it.

Initially, a thick and resistant oxide layer forms on the implant surface as a result of contact with air or physiological fluids that is closely associated with a strong osseointegration capacity, as demonstrated by the current literature. In this approach, an enhancement in surface characteristics, such as wettability or hydrophilicity, would provide greater clinical efficacy in the osseointegration process. Precisely, it has been shown that adequate surface wettability, without sacrificing the mechanical and biological properties of the surface, could stimulate osteogenic cells and promote osseointegration. On the other hand, implant surface treatment techniques, such as sandblasting and conditioning with different acids, are used to increase roughness and manipulate texture at the macro-, micro- and nanoscale.

Bone tissue has a highly dynamic behavior, in constant remodeling through cell destruction and new tissue formation, by the osteoclastic and osteoblastic cell lineage, respectively. This dynamism affects the osseointegrative process that begins from the very moment of implant insertion, continuing as long as it remains in equilibrium with the adjacent tissues. The modification of implant surfaces by different procedures, capable of producing more bioactive surfaces, stimulating greater local bone growth, has been proposed by numerous researchers, always with the aim of generating biomaterials with greater osteoinductive capacity.

With this in mind, we are excited to introduce a special edition Research Topic: “*Role of the surface in peri-implant bone biology*,” where we intend to go further on the understanding of this interaction in order to face the nowadays and future challenges concerning with the relation between the implant and the bone.

The review by Zhang et al. has developed and published the current viewpoint as well as future prospects of Advanced Polymer Processing in relation to hard tissue engineering. They expose various techniques, such as spin extrusion, electrospinning, injection micromolding and 3D printing, as well as their recent advances in the field of cell proliferation, bone repair and artificial blood vessels. In conclusion, they believe that all these novel technologies have the potential to improve and revolutionize traditional medicine as we know it.

Qiu et al. deposited a SiO_2_ layer on PEO (plasma electrolytic oxidation) based Mg implants. SiO_2_ did not alter the surface morphology, but greatly improved its corrosion resistance. Work was performed on both an *in vitro* model and a rat femur, both of which showed ample promise for osseointegration. Further study in human models will be needed to confirm all these interesting results.

Polyetheretherketone (PEEK) is a potential implant material for dental application due to its excellent mechanical properties. However, its biological inertness and poor osteoinductive ability limited its clinical application. Based on layered self-assembly, Quiu et al. decided to incorporate casein phosphopeptide (CCP) to the surface of PEEK to improve the osteoinductive capacity of these implants, by positively loading them with 3-ammoniumpropyltriethoxysilane. After that the CPP-modified PEEK specimens were studied *in vitro* and presented enhanced cell adhesion, proliferation and osteogenic differentiation of MC3T3-E1 cell, which indicate that it could be a promising strategy for PEEK implants in order to combat it biological inertness and poor osteocondutive ability. PEEK is a potential implant material for dental application due to its excellent mechanical properties. However, its biological inertness and poor osteoinductive ability limited its clinical application. Qiu et al. decided to incorporate CPP onto PEEK surface after a positive charge of 3-ammoniumpropyltriethoxysilane. After that the CPP-modified Peek specimens were studied *in vitro* and presented enhanced cell adhesion, proliferation and osteogenic differentiation of MC3T3-E1 cell, which indicate that it could be a promising strategy for PEEK implants in order to combat it biological inertness and poor osteoconductive ability.

Finally, Nansi López-Valverde et al. presented a preliminary preclinic study, in which they aimed to evaluate the effect on osseointegration of dental implants surface-treated with carboxyethylphosphonic acid and functionalized with BMP-2. They used sixteen implants that were randomly inserted into the tibiae of minipigs. Eight of the sixteen were titanium implants with a conventional etched surface (SLA type) -control group- and the other eight experimental implants were treated with CEPA and BMP-2. After a period of 4 weeks, they evaluated five bone parameters: bone in contact with the implant, bone in contact with the corrected implant, new bone formation, bone density between threads and peri-implant bone density.

In summary, and despite the fact that implant surgery is a common technique among dental clinicians, reliability and durability, and ultimately osseointegration, remain daily challenges. Further research into implant surface treatments, that further improve clinical outcomes is necessary and justifiable.

